# Effects of Cervical Rotatory Manipulation (CRM) on Carotid Atherosclerosis Plaque in Vulnerability: A Histological and Immunohistochemical Study Using Animal Model

**DOI:** 10.1155/2019/3793840

**Published:** 2019-02-04

**Authors:** Ji Qi, Ruiyue Ping, Shaoqun Zhang, Yanxiao Xu, Kai Wu, Yikai Li

**Affiliations:** ^1^School of Traditional Chinese Medicine, Southern Medical University, Guangzhou 510515, China; ^2^Guangzhou University of Chinese Medicine, Guangzhou 510403, China

## Abstract

**Background:**

The safety of cervical rotatory manipulation (CRM) is still controversial, especially in patients with carotid artery atherosclerosis (CAS). The study aimed to investigate the effects of CRM on carotid plaques in vulnerability.

**Methods:**

50 rabbits were randomly divided into four groups: model rabbits with CRM [CAS-CRM (n=15)]; model rabbits without CRM [CAS (n=15)]; normal rabbits with CRM [Normal-CRM (n=10)]; and Blank-control group (n=10). CAS disease models were induced by carotid artery balloon injury combined with a high-fat diet for 12 weeks. Then, CRM technique was performed in CAS-CRM and Normal-CRM groups for 3 weeks. In the end, determination of serum level of hs-CRP and Lp-PLA2, histological analysis under HE and Masson trichromic staining, and immunohistochemical analysis with CD34 and CD68 antibody were completed in order.

**Results:**

Carotid stenosis rates on successful model rabbits ranged from 70% to 98%. The CAS-CRM group had an increased level of hs-CRP (*P*<0.05), in comparison with the CAS group, whereas effects were not significant between the Normal-CRM group and Blank-control group. In comparison with the CAS group, the positive expression of CD34 and CD68 in the CAS-CRM group increased significantly (*P*<0.05).

**Conclusion:**

CRM therapy may increase the vulnerability of carotid plaque in rabbits with severe CAS.

## 1. Introduction

Spine manipulation, one of the complementary and alternative therapies, has been increasingly popular worldwide. It has been proved effective in the treatment of many diseases [1–3]. In particular, for patients with neck pain or cervical disorder, cervical spine manipulation therapy (CSM) was considered as one of the major treatments, gradually [4, 5]. However, side effects or complications following with CSM were reported occasionally [6, 7]. Therefore, CSM aroused more concern about safety [8, 9]. The reported complications included dizziness, cervical vertebrae fracture, neurovascular injury, and even cerebrovascular accidents [7, 9, 10]. Artery dissection was considered as one of the severe complications of the CSM therapy, possibly leading to cerebrovascular disease and even death [11–13]. With regard to the severe complications, some researchers believed that the frequency was rare, with reported occurrence of 1 in 100,000 to 1 in several millions [14, 15]. However, others held the opposite opinion that a majority of them may be underreported, most probably since certain complications, such as artery dissection, could remain silent for days or weeks [16]. To date, it is still inconclusive whether CSM should be abandoned for mechanical neck pain [17].

Cervical rotatory manipulation (CRM), one of the common CSMs in Traditional Chinese Medicine (TCM), is similar to high-velocity thrust cervical techniques (HVLA) in Western medicine. On the cervical area, during the procedure, CRM always requires high velocity and low amplitude power, which could apply a tensile force rapidly along the length of the carotid vessels at the same time [18–20]. Indeed, it has been reported that CRM or HVLA may be associated with severe vertebral artery trauma and carotid artery dissection [11, 18]. It cannot be ignored that the carotid artery (CA) is in a more superficial anatomic location compared to the vertebral artery (VA), as shown by the CA pulse being easily felt on both sides of the neck under the jaw. However, previous studies mainly focused on the effects on the VA, instead of on the CA, let alone on the abnormal CA with diseases [12, 21, 22]. It is therefore important to study what effects of such manipulation would have on the CA, and even on the abnormal CA.

Atherosclerosis is one of the common vascular diseases in CA. Carotid artery atherosclerosis (CAS) would result in degradation of the collagen structure, thickening of the intima, and formation of complex plaques [23]. Furthermore, atherosclerotic plaque ruptures could induce a thrombogenic reaction and result in a thrombus, leading to cerebrovascular accidents as ischemic stroke (IS) over blood flow [23–25]. In clinic, neck pain and other cervical dysfunctions are commonly comorbid with CAS in middle-aged and senior people. Unaware of potential underlying vascular diseases, patients sometimes underwent CRM. However, little is known about whether patients with CAS would be more likely to suffer arterial complications, if they underwent CRM. Recently, the issue has been focused on. Lin et al. [26] confirmed the adverse effect of CRM on the circle of Willis and the CA with severe stenosis in hemodynamics, and Zhang et al. [27] found that the uniaxial tensile properties of rabbit arteriosclerotic CA may decrease following with CRM. However, the effect of CRM on the atherosclerosis plaque was still unknown, especially in vulnerability. Vulnerability of the plaque, the intrinsic tendency to lose its integrity, consequently induces a dramatic atherothrombotic or embolic event. Concerning the issues mentioned above, we proposed a hypothesis that CRM may increase the vulnerability of carotid plaque and even induce plaque ruptures. To evaluate the potential effect, we simulated CRM therapy on rabbit CAS model in the present study and observed the following changes in plaque vulnerability by using histology and immunohistochemistry techniques.

## 2. Materials and Methods

### 2.1. Ethics Statement

Animal care was done in accordance with the “Guide for the Care and Use of Laboratory Animals” (Office of Science and Health Reports CPRR/NIH 1996). The study protocol was performed under the approval of the Institutional Animal Care and Use Committee of China Academy of Chinese Medicine Science (No. 201506034).

### 2.2. Animals and Grouping

50 healthy male purebred New Zealand white rabbits aged three months (weight range: 2.0–2.4 kg) were housed in individual cages (China Academy of Chinese Medicine Science Experimental Animal Center, Beijing, China) and maintained at a constant temperature of approximately 21°C. Food and water were supplied ad libidum.

In accordance with random number table, 50 rabbits were randomly divided into a model group (n=30) and a control group (n=20). After CAS modeling, 30 rabbits in model group were randomly divided into two subgroups: model rabbits with CRM [CAS-CRM (n=15)]; model rabbits without CRM [CAS (n=15)]. Meanwhile, 20 rabbits without modeling in control group were randomly divided into two subgroups: normal rabbits with CRM [Normal-CRM (n=10)]; blank-control group (n=10).

### 2.3. Carotid Atherosclerosis Modeling

In the CAS-CRM group and the CAS group, CAS model was induced by balloon catheter-induced injury in the left common carotid artery (LCCA) and continued on the high-fat diet (1% cholesterol, 5% lard, 7.5% egg yolk, 86.5% common feed; Beijing Jin Muyang Experimental Animal Feed Science and Technology) for 12 weeks. Meanwhile, the rest of the rabbits in the Normal-CRM group and blank-control group continued on the regular diet (120g/day) without balloon injury for the same time.

All balloon catheter-induced injuries were performed by the same surgeon, in accordance with the previous method [28, 29]. First, the rabbits were fed with the high-fat diet (120 g/day) after adaption to the environment for 1 week. Anesthetized by intravenous injection of 22.5mg/kg 3% pentobarbital (Shanghai Chemical Reagent Co., Shanghai, China), rabbits were fixed at a supine position with the shaved neck. Second, skin cutting, blunt dissection, and surgical exposure of LCCA were performed in order. Third, 2F (0.67mm) balloon catheter (Edwards Lifesciences, CA, USA) was gently inflated and retracted, followed with repeat procedure of pushing and pulling three times inside the LCCA of each rabbit. Then, the catheter was withdrawn and the incision was sutured under asepsis. In the end, a postoperation muscular injection of ampicillin [50mg/ (kg*∗*d), five days] was given to the subjects in case of infection.

### 2.4. Ultrasonography

At the 12th week, ultrasonography examination was carried out on all rabbits. As preparation, anesthetization, fixing position, neck skin shaving, and application of warm ultrasound transmission gel were performed in order. Then, B-mode ultrasound and color Doppler images from the LCCA were obtained with a mini-probe (Philips, L15-7io) attached to the ultrasound machine (Philips, IU22). The process was completed by the same sonographer who was blind to group division and interventions, and the diagnosis of CAS was identified by previously reported methods [30, 31]. In detail, the normal LCCA showed slim, straight, and smooth arterial wall. On the contrast, the atherosclerosis was defined as the appearance of thickness and roughness of the arterial wall, protrusion into the vessel lumen, abnormal echo, and lumen stenosis. For those atherosclerotic LCCA, the maximum stenosis rate of vessel among five random positions was recorded. Meanwhile, after modeling in the CRM-CAS and the CAS groups, the rest without CAS were excluded from the rest of the procedures.

### 2.5. CRM Intervention

In the CAS-CRM and the Normal-CRM groups, CRM technique was performed on rabbits by the same experienced TCM manual therapist blinded to other procedures, at a frequency of once a day, lasting for 3 weeks, as described in previous reports [27, 32]. First, rabbit was in the sitting position, with the spine slightly flexed to relax spinal muscles fully. One of the doctor's hands was put under the mandible of the rabbit, while the other was put behind the rabbit's occiput. Second, doctors rotated the rabbits' heads in narrow arrange step by step, until the end of the range of motion (ROM). Third, at the very time when the head was rotated to its elastic barrier position, the wrenching and thrusting stress would be quickly loaded on the cervical vertebrae during the rotation, similar to HVLA manipulation [33]. The technique was applied to both sides during a single treatment. At the same time, all rabbits were carefully observed for whether manifestations of discomfort appeared, such as hemiplegia, disability, and anorexia. When the whole process of CRM intervention finished at the 15th week, ultrasonography examination was carried out again to evaluate stenosis rate of LCCA.

### 2.6. Determination of Serum Level of High-Sensitivity C-Reactive Protein (hs-CRP) and Lipoprotein-Associated Phospholipase A2 (Lp-PLA2)

When CRM intervention finished, blood samples were obtained from the medium-sized artery of ears of all enrolled rabbits. Serum was stored at -80°C until being assayed. Then, the serum levels of hs-CRP and Lp-PLA2 were measured by enzyme-linked immunosorbent assay (Elisa) of double antibody sandwich (PLAC test, diaDexus, Inc., San Francisco, CA). Analyses were performed by personnel blinded to other laboratory data.

### 2.7. Histology

Finally, all rabbits were euthanized by air embolism, and the LCCAs were harvested completely and washed with phosphate-buffered saline (PBS) buffer to remove the excess fat and connective tissues. Then, the samples were fixed in a buffered and neutral 4% formaldehyde solution, embedded in paraffin, and cut into 5 *μ*m thick sections guided by the imaging of rotary microtome (Nikon Eclipse CI). First, the qualitative analysis in plaque morphology was made on Hematoxylin & Eosin (H&E) microscopic slides in four groups. Second, in the CAS-CRM group and the CAS group, Masson trichrome staining was performed to analyze the collagen content in plaques. Quantitative analysis was performed under high-power magnification (×100), and the mean area of collagen fiber with positive staining across three random fields was determined. Each section was measured three times, and the averaged results were reported, by use of the Image-Pro Plus 6.0 software (Media Cybernetics, Inc., Rockville, MD). Collagen content was presented as the ratio of the area of positive staining to that of the whole plaque.

### 2.8. Immunohistochemistry

In the CAS-CRM group and the CAS group, sections were also prepared for immunohistochemistry techniques in accordance with the previously described method [34–36]. Specimens were incubated with 1:100 dilutions (phosphate-buffered saline, PBS PH 7.4) of antibodies (Abcam®) overnight at 4°C against CD34 or CD68 and then washed and incubated with their corresponding secondary antibody conjugated to biotin for 20 m and rinsed in buffer (PBS PH 7.4). The sections were counterstained with hematoxylin, rinsed, and cover-slipped. For negative immunostaining controls, the primary antibody was replaced with PBS. Chromogenic immunohistochemistry slides were imaged with an Eclipse CI microscope (Nikon; Apidrag, Romania) equipped with a 5 Mp CCD camera and the Nikon Nis Elements software. Sections were photographed with the same bias light under high-power magnification (×200), and the mean number of macrophages and endothelial micro-vessels in plaques with positive staining was counted across five random microscopic fields, respectively. In detail, the mean number of endothelial micro-vessels was counted by mean micro-vessel densities (MVD), and of macrophages by the integrated optical densities (IOD). Microscopic analysis was performed by use of the Image-Pro Plus 6.0 software (Media Cybernetics, Inc., Rockville, MD).

### 2.9. Statistical Analysis

Data were presented as the mean and standard deviation (SD). Two-way factorial ANOVA was used to determine the effects of CRM and CAS modeling, and the Bonferroni method was then used for assessing differences, in serum level of hs-CRP and Lp-PLA2 among the four groups. Paired-samples t-test was used to compare the carotid stenosis rate before CRM and the one after CRM within each group. Differences between the CAS-CRM group and the CAS group in carotid stenosis rate, ratio of collagen fiber, MVD, and IOD were compared by independent-samples t-test. A value of* p*<0.05 was considered significant. All analyses were performed by SPSS 20.0 software (IBM, Armonk, New York).

## 3. Result 

### 3.1. Animal Situation

**Figure 1:**
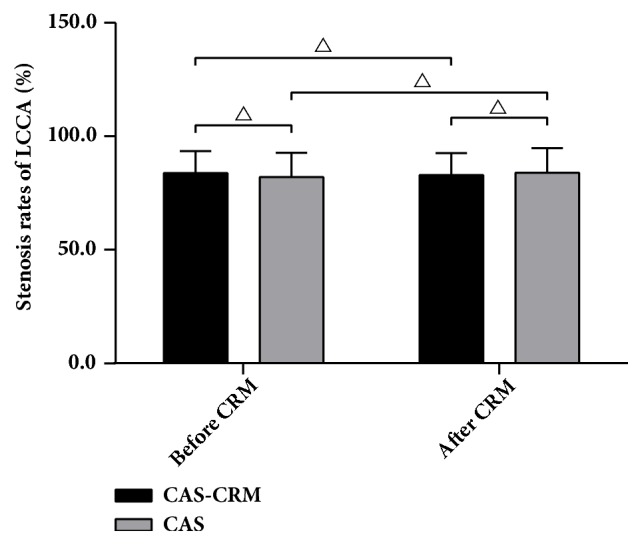
*Stenosis rates of LCCA in the CAS-CRM group (n=8) and the CAS group (n=10) (mean, SD).* ^△^ *P*>0.05;* CRM*, cervical rotatory manipulation.

A total of 19 rabbits with typical CAS were included: 9 in the CAS-CRM group and 10 in the CAS group. However, another rabbit in the CAS-CRM died due to diarrhea during the process of CRM intervention. Stenosis rates of rabbits LCCA in the CAS-CRM group and the CAS group ranged from 70% to 98%. No significant difference was found in carotid stenosis rates between the CAS-CRM group and the CAS group, either before or after CRM intervention (*P*<0.05). In addition, no significant difference was found, comparing the stenosis rate before CRM and the one after CRM within each group (*P*>0.05) (Table 1; Figure 1).

### 3.2. Serum Levels of hs-CRP and Lp-PLA2

**Figure 2:**
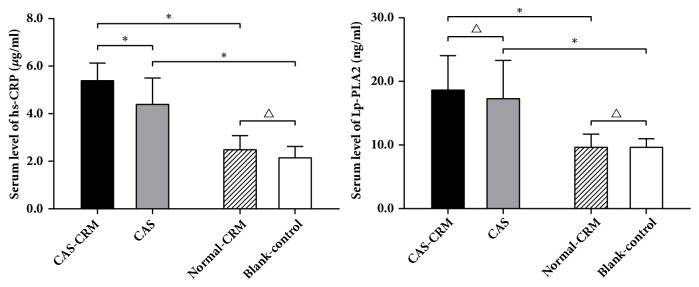
*Serum level of hs-CRP and Lp-PLA2 in four groups (mean, SD). ∗P*<0.05; ^△^*P*>0.05.* CAS*, carotid atherosclerosis;* CRM*, cervical rotatory manipulation.

In the serum level of hs-CRP, no interactive effect was significant between CRM and CAS modeling (*P*>0.05), while main effects of CRM (*P*<0.05) and CAS modeling (*P*<0.05) were significant. Multiple comparisons showed that the level of hs-CRP in the CAS-CRM group was higher than that in the CAS group, significantly (*P<0.05*). However, no significant difference was noticed between the Normal-CRM group and the Blank-control group (*P>0.05*) (Table 2, Figure 2).

In the serum level of Lp-PLA2, no interactive effect was found between CRM and CAS modeling significantly (*P*>0.05). Significance was found in the main effects of CAS modeling (*P*<0.05), but not in CRM (*P*>0.05). Multiple comparisons showed that no significant difference was found between the CAS-CRM group and the CAS group (*P*>0.05). Similarly, no significant difference was found between the Normal-CRM group and the Blank-control group (*P>0.05*) (Tables 2 and 3, Figure 2).

### 3.3. Histology

**Figure 3:**
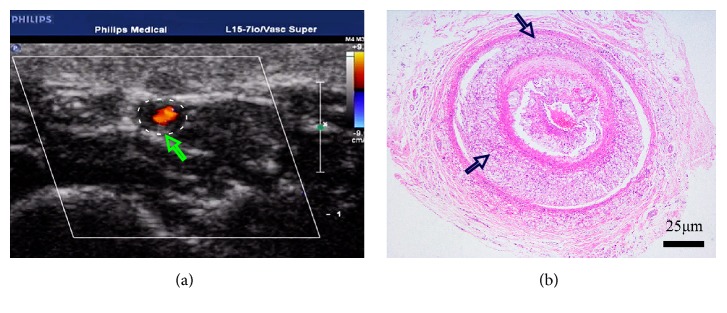
*Characteristics of CAS in the CAS-CRM group and the CAS group.* (a) Lumen stenosis, thickening of the intima, and plaque in cross-section image under ultrasonography (arrow). (b) Accumulation of foam cells, enlarged area of cell discoloration, disordered cell arrangements, and fuzzy structures (arrows) (HE stains, ×40).

**Figure 4:**
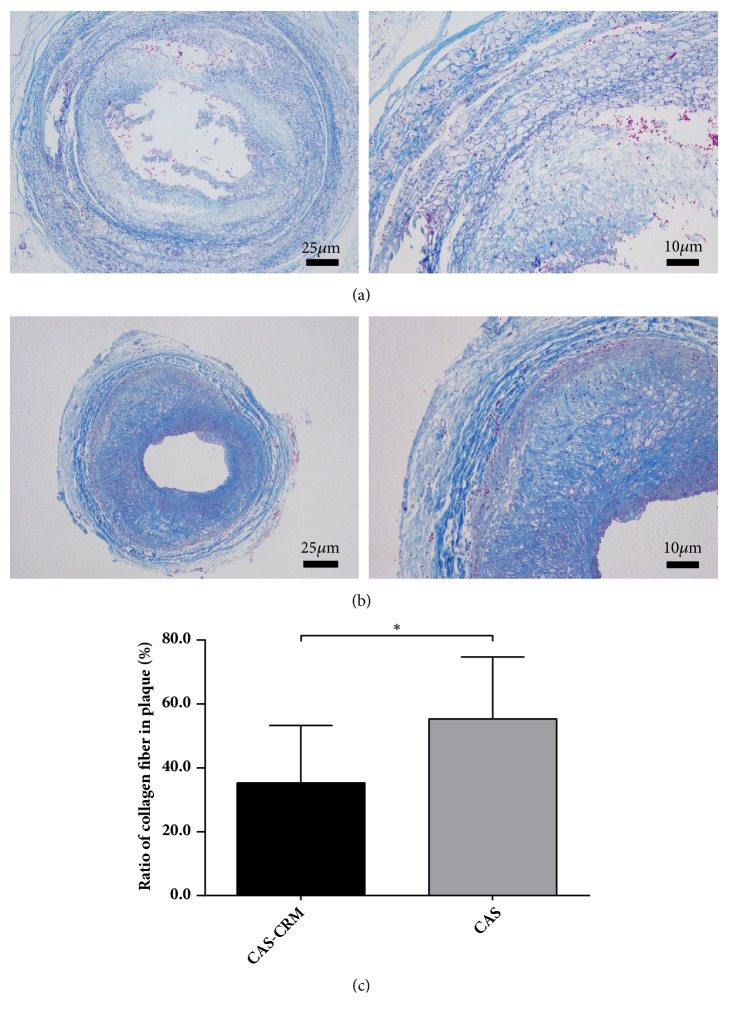
*Histological characteristics of collagen fiber within carotid plaques in the CAS-CRM group (n=8) and the CAS group (n=10) (Masson stains; ×40 and ×100, respectively).* (a) CAS-CRM group. (b) CAS group. (c) Difference in ratios of collagen fiber in plaque between the CAS-CRM groups. *∗P*<0.05;* CAS*, carotid atherosclerosis;* CRM*, cervical rotatory manipulation.

In the CAS-CRM group and the CAS group, characteristics of CAS were found under both ultrasonography and histology. First, lumen stenosis, thickening of the intima, and carotid plaque were visualized in cross-section image under ultrasonography. Second, in HE stained slides, accumulation of foam cells, enlarged area of cell discoloration, disordered cell arrangements, and fuzzy structures were identified in models (Figure 3). Besides, in Masson stained slides, layered collagen fibers were observed. Microscopic analysis revealed that the CAS group had a lower level of collagen fiber in plaque than the CAS group, significantly (*P*<0.05) (Table 4, Figure 4).

### 3.4. Immunohistochemistry

**Figure 5:**
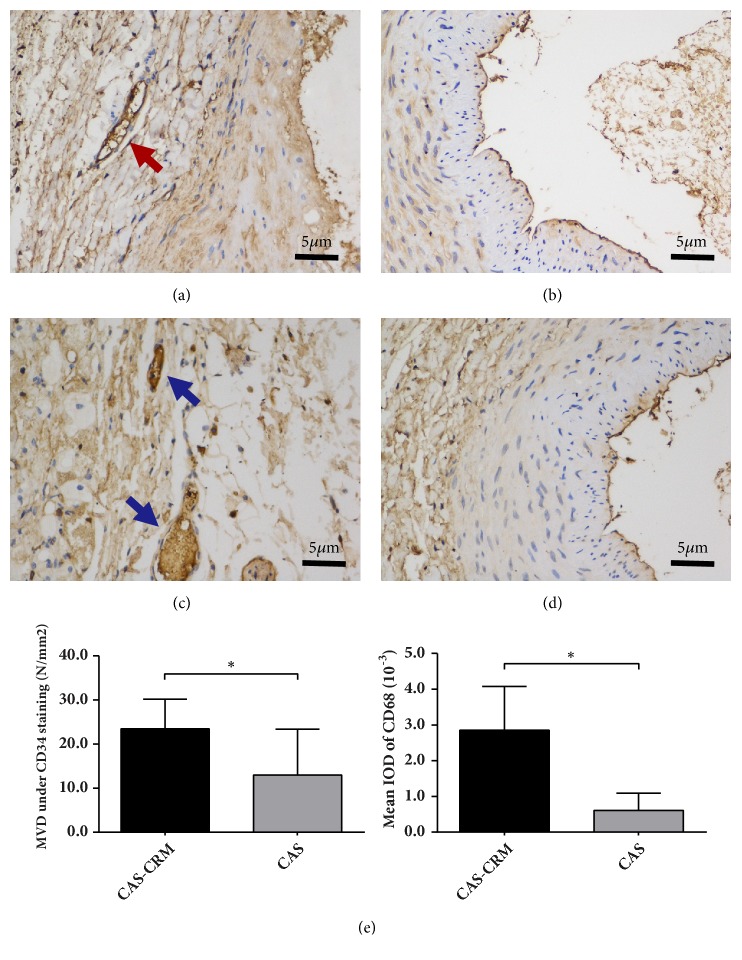
*Immunohistochemistry of CAS in the CAS-CRM group (n=8) and the CAS group (n=10).* (a) CD34 staining in the CAS-CRM group, micro-vessel with brown stained (×200) (red arrow). (b) CD34 staining in the CAS group (×200). (c) CD68 staining in the CAS-CRM group, macrophages with brown stained (×200) (blue arrow). (d) CD68 staining in the CAS group (×200). (e) Difference in MVD and IOD in plaque between the CAS-CRM and the CAS groups. *∗P*<0.05;* CAS*, carotid atherosclerosis;* CRM*, cervical rotatory manipulation;* MVD*, mean micro-vessel density;* IOD*, integrated optimistic density.

In immunohistochemistry staining slides, micro-vessels or macrophages appeared to be brown in positive expression of CD34 or CD68, respectively. Microscopic analyses revealed that MVD and mean IOD of macrophages increased significantly in the CAS-CRM group, in comparison with those in the CAS group (*P*<0.05) (Table 4, Figure 5).

## 4. Discussion

This study aimed at exploring the effect of CRM therapy on CAS. However, it is difficult to carry out the study in the human subjects, due to ethics limitation and safety consideration. Meanwhile, animal model was chosen for several reasons. First, CAS model of rabbit was previously confirmed to be available for replicating CAS in human body [28, 29], as atherosclerotic lesion in rabbit is similar to those in human. Second, the rabbit CA is superficial and straight, which helps to reflect the similar effects of CRM on CA in human body. Third, balloon injury was a common and efficient method to induce CAS model. In the current study, we found that using a smaller catheter helps to decrease the occurrence of the rupture of rabbit carotid artery during the operation. Taking the success rate of modeling into consideration, we included 5 more rabbits in the CAS-CRM group and the CAS group, respectively, to ensure the suitable sample size in the following tests and comparisons. Besides, the application of ultrasonography improved efficiency in evaluating the existence of carotid plaque, as there was no need to observe characteristics of vascular samples in vitro. Finally, the following histological findings confirmed the successful development of CAS model. In fact, all carotid stenosis rates on successful model rabbits ranged from 70% to 98%, which appeared to be classified as severe CAS (stenosis rate: 70%-99%), in accordance with the related diagnostic principle [37].

In atherosclerosis disease, plaque vulnerability is the key to predict the risk of cerebrovascular incidents. A vulnerable plaque is unstable and prone to rupturing. Multiple methods have been confirmed to be efficient in predicting the vulnerability of the plaque [24, 26, 27, 30, 38–40]. In the present study, the Elisa inspection of serum inflammatory biomarkers, histological stain on the component of plaques, and immunohistochemistry methods were combined together, which possibly had higher specificity in predicting and evaluating the effects of CRM on carotid plaques in different aspects.

It is well known that inflammation plays a key role in the pathophysiology of atherosclerosis, from endothelial dysfunction through all stages of plaque build-up to its detrimental clinical ischemic complications [41, 42]. Of the dozens of candidate biomarkers, hs-CRP and Lp-PLA2 have been demonstrated as strong predictors of incident cardiovascular event, with enough accumulated study evidence. Hs-CRP is a classical acute phase protein, represents a clinical marker of inflammation, and is closely associated with the development of inflammation in AS plaque. Lp-PLA2 is produced by macrophages and foam cells in the vascular intima in atherosclerotic plaque, and its biology is linked to the causal pathway of plaque inflammation and ultimate rupture. The serum levels of hs-CRP and Lp-PLA2 are not only risk predictors, but also the key in risk-guided therapy of atherosclerosis diseases [43–46]. This study found that the main effects of CAS modeling on the serum levels of hs-CRP and Lp-PLA2 were significant. That is, CAS group had a significantly higher level of both serum biomarkers than Blank-control group, and a similar difference was found significantly when comparing the CAS-CRM group and Normal-CRM group. The result was consistent with the findings of related researches, which probably indicated the inflammation involved in the development of CAS and the occurrence of plaque rupture [47]. Moreover, the serum level of hs-CRP in the CAS-CRM group was higher than that in the CAS group, significantly. However, there is no significant difference between the Normal-CRM group and Blank-control group. These findings indicated that adverse effects of CRM, especially in inflammation, were more significant in rabbits with CAS than the normal. The elevated serum level of biomarkers following CRM in CAS rabbits possibly reflected the increased inflammation in carotid plaques, and it seems to be one sign of development and rupture of plaques.

Besides involved inflammation, morphology of atherosclerosis plaque is another important factor affecting the vulnerability of plaque. As the previous study proved, formation of thromboembolism is related not to plaque size but to plaque instability, with increased embolization occurring at ulcerated plaques and thrombosis associated with plaque rupture [48]. Hence, the risk of rupture depends not only on the thickness of the plaque's fibrous cap but also on the mechanical properties of the artery wall and plaque components. Naghavi et al. [49] and Virmani et al. [50] have shown that a vulnerable or unstable plaque is typically defined by a large extracellular necrotic core (≥25% surface area) shielded from the blood by a thin fibrous cap infiltrated by macrophages. In a word, the plaque vulnerability mainly depends on the content of lipids, macrophages, and collagen [51]. In this study, we also found that histological and immunohistochemical characteristics of carotid plaques in the CAS-CRM group showed specificity following CRM intervention. First, in Masson staining, it was observed that the content of collagen fibers in arterial plaque was significantly lower in the CAS-CRM group, in comparison with the CAS group. Previous research results have shown that the decrease of collagen plays an important role in increasing the risk of plaque rupture [51, 52]. Second, in CD34 and CD68 staining, micro-vessels and macrophages were observed in plaques. Also, infiltration of macrophage cells, plaque hemorrhage, and even neovascularization with increased micro-vessels were seen as the important indicators of vulnerable plaque [48–50]. Further, immunohistochemistry result revealed that the MVD and IOD of infiltrated macrophage cells were both significantly higher in the CRM-CAS group than those in the CAS group (*P*<0.05). In accordance with the above-mentioned findings, the results could demonstrate that the vulnerability of rabbit carotid plaque showed a significantly increasing trend following CRM, decreased fibrous, and increased infiltrated macrophages and neovascularization.

As one of the most common TCM therapies, CRM has been gradually involved in related studies [27, 30]. Although the decreasing effect on mechanical properties of rabbit atherosclerotic carotid artery has been confirmed previously [30], the mechanism of the effect has not been focused on. It is known that the variation in mechanical properties of the atherosclerotic artery is associated with the variation in component and structure of plaque and artery [53]. The above result could provide an explanation in histology. That is, CRM may destroy the original vascular structures of rabbit atherosclerotic CA and thereby result in degeneration of mechanical properties of the artery. What is more, both degeneration of mechanical properties and the above histological changes indicated the increasing vulnerability of atherosclerotic plaque.

Nevertheless, this study still has some limitations. First, animal models were finally confirmed to be severe CAS, and the effects of CRM on mild or moderate CAS were still unknown. Second, CRM technique was carried out by the same therapist to standardize the intervention as far as possible; however, CRM used on animals is only similar to CRM used on human body, but it is not the same thing. Third, the study found no significant difference in serum level of Lp-PLA2 between the CAS-CRM and the CAS group, possibly attributing to sample size, measurement error, and so on. In addition, the common clinic-treatment course of CRM was performed in the study, and it was still unknown whether the effects were associated with the frequency of intervention or not. All the issues remain to be solved in the following studies.

## 5. Conclusion

In conclusion, we found that CRM therapy may increase the vulnerability of carotid plaque in rabbits with severe CAS. However, it remains to be explored in the future whether this therapy would have the same effects on human subjects. Although the exact effect on the human subjects is unknown, the results of this animal study raise further questions about the application of CRM in the presence of known or suspected atherosclerotic plaques.

## Figures and Tables

**Table 1:** Stenosis rate of rabbits LCCA in the CAS-CRM group and the CAS group (mean±SD).

Group	N	Before CRM	After CRM	*P*
CAS-CRM	8	83.81±9.64	82.88±9.70	0.076
CAS	10	82.08±10.61	84.00±10.86	0.151
*P*		0.725	0.822	

**Table 2:** ANOVA on fitted model for the effect of CAS modeling, CRM, and their interaction on the serum level of hs-CRP and LP-PLA2.

	hs-CRP					Lp-PLA2				
	SS	*df*	*MS*	*F*	*P*	SS	*df*	*MS*	*F*	*P*
CAS modeling effect	62.685	1	62.685	104.845	<0.001	651.266	1	651.266	37.782	<0.001
CRM effect	4.220	1	4.220	7.058	0.012	4.114	1	4.114	0.239	0.628
CAS modeling × CRM interaction	1.061	1	1.061	1.775	0.192	4.473	1	4.473	0.259	0.614
Error	20.328	34	0.598			586.075	34	17.237		
Total	85.773	37				1237.963	37			

*CAS*, carotid atherosclerosis; *CRM*, cervical rotatory manipulation; *SS,* sum of squares; *df,* degrees of freedom; *MS,* mean square.

**Table 3:** Multiple comparisons in the serum level of hs-CRP and LP-PLA2 among four groups (mean±SD).

Group	N	hs-CRP (*μ*g/ml)	Lp-PLA2 (ng/ml)
CAS-CRM	8	5.39±0.74^a,c^	18.63±5.41^a^
CAS	10	4.39±1.11^b^	17.28±6.02^b^
Normal-CRM	10	2.48±0.60	9.63±2.08
Blank-Control	10	2.14±0.48	9.65±1.34

*CAS*, carotid atherosclerosis; *CRM*, cervical rotatory manipulation.

Two-way factorial analysis of variance results:

a denotes* P*< 0.05 significance vs. Normal-CRM group;

b denotes* P*< 0.05 significance vs. Blank-control group;

c denotes* P*< 0.05 significance vs. CAS group.

**Table 4:** Histological characteristics of carotid plaques in the CAS-CRM group and the CAS group (mean±SD).

Group	N	Ratio of collagen fiber (%)	MVD (N/mm^2^)	Mean IOD(*∗*10^−3^)
CAS-CRM	8	35.31±18.02	23.43±6.74	2.85±1.23
CAS	10	55.36±19.36	12.97±10.39	0.62±0.48
*P*		0.039	0.026	0.001

*CAS*, carotid atherosclerosis; *CRM*, cervical rotatory manipulation; *MVD*, mean micro-vessel densities; *Mean IOD*, mean integrated optical densities.

## Data Availability

The research data used to support the findings of this study are available from the corresponding author (Yikai Li) or the first author (Ji Qi) upon request.

## Supplementary Materials

Supplementary Materials Initial data of rabbit carotid stenosis rate, the serum level of hs-CRP, the serum level of Lp-PLA2, and the ratio of collagen fiber, MVD, and IOD in the present study.
